# Research by autistic researchers: an “insider’s view” into autism. The autistic way of being

**DOI:** 10.3389/fpsyt.2025.1664507

**Published:** 2025-10-22

**Authors:** Wenn Lawson

**Affiliations:** Occupational Therapy in School of Allied Health, Curtin University, Perth, WA, Australia

**Keywords:** adaptive functioning, autism spectrum, inclusion, neurodiversity, neurodivergence, social cognition

## Abstract

This paper introduces us to an increasingly popular understanding of autism, but as understood and experienced by Autistic people: the descriptive theory of Monotropism. Initially this paper sets out the background to monotropism as the author briefly mentions various autism theories and highlights some reasons why such theories didn’t resonate with Autistic people. Uncovering how monotropism explains autism and Autistic experience takes the reader into the very heart of Autistic experience in ways not previously shown. As well as discussing attention, interest and connections to the Autistic sensory experiences (external and internal) the author highlights the experience of Object Permanence (OP) and its impact upon Autistic lives. Current research is beginning to demonstrate that OP is experienced differently in Autistic lives to non-autistic lives. Initially though Autistic people were thought to relate to OP in similar ways to non-autistic people (e.g.), although there were hints that aspects of OP in Autistic people were experienced differently. The reasons for why monotropism has grown in popularity and how this theory explains the Autistic experience are set out below.

## Introduction

Research should be conducted in a noble, honourable, and respectful way that explores a topic or subject area via thorough, structured and thoughtful means. Ideally, if the research concerns a particular community, then members of that community should be involved. The purpose of the research isn’t to only uncover lots of facts about the topic, although that is important, but it’s to show and share, in an accessible manner, how those facts translate to increase our understanding of an individual’s (human) life and, if necessary, what action(s) might be required because of the research.

As echoed above in the abstract, OP features much more promanantly in Autism than previously thought (1–3). Where autism is concerned there have been many theories as to its cause and many research ideas have sought to explain Autistic behaviour. But these were always from the viewpoint of someone looking at autism and Autistic people ‘from the outside’ and via the lens of typicality. Initially autism wasn’t considered as any type of spectrum or that it might exist in women (4). It was thought of as a rare, male, mental disorder that either occurred with high rates of intellectual disorder or, if found in persons with relative intelligence, these people were odd and emotionally distant (4–7). Eventually though, our understanding of autism, due to research over time and especially over the last decade, changed. The idea of neurodiversity (all minds and neurological processing styles are of value) arrived (8–13).

The journey towards the concept of neurodiversity (all brains are different and contribute to humanity) has been fraught with obstacle after obstacle and much of the resistance seems to have been imbedded in the nature of the power dynamics (academics, university’s funding and so on). By this I mean the academic fervour and the race to own new discoveries can override what is actually apparent, and it takes practice, funding and strategy to get past this (14). However, with the changes attributed to ideas of equality, listening, democracy and Co-production (See: Autism CRC: https://www.autismcrc.com.au/ Autistic people have taken the centre place on the platform and have raised their flag; above academia, the halls of power and despite the challenges around funding!

The one theory voiced, experienced and researched by autistic people, is the theory of monotropism (15). Monotropism means (mono) single focus and tropism (channels) implies attention. So, monortropism means single focussed attention. This theory is more descriptive (currently) and is often attributed to areas of captured attention, interest (positive or negative) and the human experience of living with difficulty shifting attention, sensory differences because of this and often literalness in communication (may not get the joke or perceive the subtleties of the hidden curriculum of human communication). These are the main attributes associated with autism and some aspects of Attention Difficulties with or without Hyperactivity known as ADHD (16). This is the theory that has stood the test of time and is echoed by autistic people (e.g. 1, 2, 15, 17–22). The author believes this theory explains all the aspects experienced by Autistic people. Monotropism also has gained credibility among the Autistic community and is supported by current research (e.g. 17; 25, 20, 2012, 15). Of course Autism and Autistic expression is influenced by each Autistic person via their own personality, background, intersectionality and history. Many personal factors impact the Autistic person and the monotropic lens through which their Autism is experienced. This is explained more in the sections below.

## Autism theories (the journey)

Many theories exist as to why autism impacts peoples’ lives the way it does. Theories that tried to find the cause of autism ranged from ‘refrigerator mothers’, (cold, distant and uninvolved parents) (6, 23) to eating gluten (24). These ideas were not substantiated over time and have been debunked. We had theories that said Autistic people ‘lacked a theory of mind’, but too many Autistic people passed tests for ‘Theory of Mind’ (ToM) See: Baron-Cohen et al. (25)Frith (26) and Jones et al. (27). Also for an updated summary see: Happé, and Frith, (28). When a more complex test was introduced for ToM, (29) it showed Autistic adults passed certain aspects of ToM but failed others. However, the authors concluded that ToM was also something learnt via experience and over time, implying issues with ToM were not specific to nor causative of autism. Other’s theorised autism was about problems with ‘executive dysfunction’ and a lacking in capacity to gain a central cohesive picture (30, 31), see also Happé, and Frith (28). But difficulties with executive functioning are not only seen in autism, but in various other human challenges (depression, ADHD, personality differences, SLD and so on) e.g. see: Benallie, et al. (32) El Wafa et al, (33) Sharifi and Asanjarani (34). 35).

Then there were theories that said as Autistic people we had ‘enhanced perceptual functioning’ abilities; at least this one noted strength, and not just deficits (36). Some argued autism was said to be associated with having a more ‘male brain’ (37) but many Autistic individuals disagreed with this (20). Also, researchers worried about this gender bias as being problematic and contributing to poor diagnosis of Autistic females (e.g. 38). In many ways some of the above had aspects of ‘truth’ in them but essentially, they missed two things: No-one asked or listened to the experience of Autistic people; we had no say in what professionals announced as ‘Autism’ and secondly, they were all deficit based and disease motivated. The current textbook for various ‘mental disorders’, the DSM-5 (2013:2022) clearly states autism is a ‘developmental disorder’ implying it changes over time, it is not a disease. However, many professionals still view autism in pathological terms and as a mental disorder impacting negatively cognitive processing rather than a difference of cognitive processing (e.g. 39). This author argues when monotropism is considered as a way of experiencing the world, we can remove the pathological lens. The author also concedes that when autism isn’t understood and appropriate accommodations for individuals are not made, then pathology will result.

It’s also the case that intellectual and cognitive challenges, learning difficulties and others, can co-occur with autism, but are not autism. Thankfully, over time the way we are thinking about autism has begun to change, and Autistic people are advocating for themselves. We began to write books and tell the world what we experienced (18, 40–42). We wrote about difficulty doing more than one thing at any one time, and of becoming easily overloaded or of being very ‘mono’ (18, 41, 42). We cited the differences with our sensory systems, both external and internal (21, 40, 43, 44). However, only the later version of the DSM-5 (2013) mentioned sensory differences in autism, these were not mentioned in earlier versions of the text. We also wrote about our strengths, often based in flow states and of becoming experts in our field of interest (an attribute of being very mono) ( 17, 18, 45–47).

Many fields of employment and career areas noted the high population of Autistic people (NASA, The Military, Education, Engineering, The Arts, the World of Music, The Police Force, The Medical Profession and so on) See: are-on-the-autistic-spectrum within their ranks. It was agreed that autism often equated with the ability to be single minded (monotropic) and good at work environments that were structured (46). But Autistic people also seemed to have a higher than ‘expected’ pain threshold (I didn’t notice pain that I was expected to when I broke my elbow) (possibly due to being elsewhere focussed with no spare attention to notice pain) and were also more susceptible to autoimmune and inflammatory ailments (18, 48–50). Monotropism might play a role here but has not yet been researched. Being mono will impact dietary habits, exercise regimes and general self care.

## Monotropism or single focussed attention

Eventually the concept of monotropism; having single focussed attention, (an interest system of mind) was captured by the minds and work of three Autistic people: Murray et al. (15), who had been working on this understanding since the early ‘90’s. Although it has taken a very long time (autism was first noted in the late 1930’s and early 1940’s) the world appears to finally be listening to the voices and experiences of Autistic people (9, 22, 46, 51). Over much of the last decade, there has been a steady uptake by Autistic and non-autistic researchers to examine this theory and develop a tool to explore it (e.g. 17, 22, 47, 52). See also Monotropism.org for a fuller exploration of the literature on monotropism.

Everyday life, from communication within social interaction and societal expectation, interoception experience, outer sensory experiences and those of Object Permanence impact differently upon Autistic people than they do for non-autistic people (21, 53, 54). I suggest these are all aspects of being monotropic. All too often though this is overlooked. Research for all humans is often compared to what is called ‘normative data’. The issue is, normative data for who? We might take a group of eighteen-year-old males and explore the variability of height to obtain the average, but the average height will be different for different cultures, race and ethnicity, as well as how they are affected by diet, weather systems and education. Simply stating the average height for male eighteen-year-old college students in The Netherlands will not translate to the average height of eighteen-year-old college students in Mexico. So, comparing Autistic people with non-autistic people is like comparing walnuts with elephants! Apparently, I can’t use the term ‘comparing apples to oranges’ as these fruits have 12 out of 15 elements in common (55). I was going to argue that although apples and oranges are both a type of fruit, they look different, feel different, taste different and have differing compounds in their make-up!

What we have come to understand about monotropic processing styles is that being mono spills over and into every other aspect of our lives (18, 41, 56 See: https://www.youtube.com/watch?v=wOe1fliDs0I). The DSM 5 (2013:22) mentions ‘sensory differences’ in autism. It doesn’t go into lots of detail only stating: ‘Hyper- or hypo-reactivity to sensory input or unusual interests in sensory aspects of the environment (e.g., for apparent indifference to pain/temperature, adverse response to specific sounds or textures, excessive smelling or touching of objects, visual fascination with lights or movement). However, the author and other Autistic individuals believe these sensory differences are directly based in monotropic processing (16, 20).

We all have an internal and external sensing system. Our external system of sensing is well-known. We have ‘sight, hearing, tasting, touching, smelling, movement and balance’. For many Autistic people one, two or more of these senses can appear as overwhelming or underwhelming, (e.g. noise is too loud/not loud enough, sight is too bright/too low, touch can be experienced as ‘sudden’ and/or as pain/or not noticed, taste textures can feel uncomfortable/constant need for oral stimulation, movement and balance can be experienced differently, as in circling round and round but not feeling dizzy when you stop and much more) (18, 21, 41). The above are directly related to our Autistic experience of being monotropic, experiencing one sense at any one time can heighten or dilute it.

## External sensing

Monotropism asserts Autistic external sensing is more likely to be experienced as the heightening of one sense over another and not diluted as it doesn’t get shared across the senses, or under felt/noticed, due to no/little attentional capacity (20). When attention is all consumed with whatever the individual is attending to, there is no spare attention for anything else. So, if one’s sense is taken up with what it sees, hears, tastes and so on, it can only be taken up with that one sense/thing. If attention is occupied by a specific interest (trains, reading, fashion, water, power-lines, pain, discomfort or comfort and so on) it may fail to attend or notice other external senses or it may mean experiencing being ‘satisfied with a non-satisfying satisfaction’! If this is the case, it means I need to constantly seek out more.

## Interoception, or our internal sense

Interoception is our eighth sense. It refers to our perceptions of what is going on inside our bodies. Interoception is responsible for feelings of hunger, thirst, sickness, pain, having to go to the bathroom, tiredness, temperature, itch and other sensations that originate from internal sensing. Interoception helps develop our connection to self, as when we understand how we feel, we learn to connect to ourselves and connect to others (53). Inside us, our body is constantly sending interoceptive messages to our brain so our brain can inform us if any action is needed (e.g. if our bladder is full, we need to empty it; if we are hungry, we need to eat and so on). For Autistic individuals, who are monotropic (all their attention is focussed on what has captured their interest) however, there may be no spare attention to ‘notice’ our interoception sense. This means we might not recognise when we need the bathroom or when we are hungry. So, all of us need to notice the signals from our interoception system of sensing but, if we are autistic, we may need support to do this. Noticing and regulating our emotions is also connected to our interoception system of sensing. This means, when we are not noticing our interoceptive signals it impacts our ability to self-regulate. A good video to watch to explain interoception: https://www.youtube.com/watch?v=esnhrnEotwg See also Barret and Quigley, (57).

Neuroception, is the unconscious sense enabling us to notice danger (being with a safe human (a care-giver) or a potential non-safe human (a stranger). Also, this internal body state of safe or dangerous, triggers neurobiologically determined prosocial or defensive behaviours (e.g. knowing I’m about to throw-up, hit out at someone or something and so on).

Interoception and neuroception are linked because they each inform the other (58). But, in autism, when our attention is taken over by a captured interest (joyful, painful, sad, exciting, some ruminating thought and so on) we may not notice or be able to name, either inner or outer sensory information (17, 22).

Neuroception is the autonomic, unconscious sense that warns the body of danger (Subconscious-System-for-Detecting Porges/7aa83dc8d507fc38aa97e22233d96fd878ff7e51). It allows a person to connect to the five F’s (Fear, Flight, Freeze, Fight and Fawn). In autism, however, this system will be skewed, meaning we are primed for anxiety and possible hyper vigilance, hypo vigilance or disconnection (17, 22, 59). Although interoception is a sense, it is different from noticing our external environment as it lets us connect to our internal world (as above but also heart rate, breathing, temperature, appetite, thirst, sexual desire and others), rather than the external world (sight, sound, touch, taste, smell, movement).

Interoception is a sense that is often neglected though, not just in terms of academic research, but as human beings, we may not realise how important interoception is (53). We need a good interoceptive sense that is fine-tuned, otherwise we may not have access to ‘self-regulation’ of our emotions, behaviour or of our actions. As we understand the role monotropism plays in our sensing (external and internal) we can appreciate why we find difficulty with heightened or underwhelmed senses. Then we can begin to explore the need for finding ways that help us, as Autistic people, connect to our interoceptive selves (See: regulation/get-ready-to-learn/Many of the exercises recommended in the link for children and young people also work for adults.

Monotropism, therefore, may explain ‘pica’ (the putting of non-edibles into the mouth) and some food avoidance (fussy eating) as well as the need for more ‘saltier, sweeter, stronger tasting foods, and so on. It might also explain why our interoceptive ‘noting’ of internal messages about hunger, thirst, pain, being unwell or well and so on are missed.

Object Permanence (OP) Although it shouldn’t, being respectful of autistic features may depend upon better understanding of autism and neurodivergence. In Autism we often see difficulties in sensory-motor-cognitive-emotional integration: passing from single tasks to multiple coincident tasks. Knowing why this is so could help promote better communication, which would be one tool in salvaging the ‘double empathy’ problem (see: 60, 61). To help with this, knowing why in autism we prioritise our attention towards focussing on specific things (we are monotropic) and why we may have issues with attention maintenance, could help solve this puzzle. Our desire for order and predictability can look like obsessive-compulsive disorder, but our drive for similar, compared to different ways of doing something, comes from a different place and for different reasons; it’s because we are mionotropic.

What is OP? Object permanence is the understanding that things continue to exist even when we can’t see or sense them. It’s like knowing that your favourite book is still in your room even when you’re not there to see it. But this doesn’t just apply to objects, I think it applies to people, seasons, clothing, animals, expectations, plans and so on (22).

For most people, this understanding develops when they are babies (https://www.simplypsychology.org/object-permanence.html. However, if you’re autistic, you might experience object permanence differently, which can affect how you perceive both people, objects and other things around you.

## How OP might affect an autistic person

As an autistic individual, object permanence differences might show up in our lives in several ways, cognitively, emotionally and physically. But, if these are not understood, they can be thought of as an individual being deliberately rude, indecisive, insensitive, difficult and uncaring (1:^2^) or lacking in ToM. Emotionally, an Autistic individual might overreact to a situation or not react in ways expected. Being worried when a familiar item is lost or removed can cause extreme panic and ‘melt down’. Then again, a person can experience death of a close relative or friend and seeming not react at all until the usual routine or way of being with that person fails to happen. A change of routine or someone simply leaving the room can cause incredible distress. If OP is poorly formed, then forward thinking will be difficult too. This is one reason the ‘now’ and ‘next’ cards or ways of explaining can support us in our daily lives. Being monotropic and only focussed on ‘the now’ can prevent us from appreciating outcomes or from forward thinking.

Finding, establishing and maintaining relationships can be difficult to navigate for similar reasons. If you are not a numbers person you may find it difficult to remember birthdays and special occasions. But even if you do remember you might not think to do anything about it. Texting, phone calls and personal encounters help to further friendships but knowing this and doing it can be really difficult for some Autistic people with poor OP. Sometimes the anxiety at being separated from caregivers or loved ones can be extreme or not noticed. At other times it can feel that a person has ceased to exist if they are out of sight. This, I believe, is connected to school refusal in some Autistic children: ‘I can’t see my dog, how can I know they are safe at home? I need to be home to check’. At times for some Autistic people with poor/low OP, going shopping, or to some event, can be hard to comprehend, especially if there are changes from a previous time they did this. If one shop or expectation changes it feels like the whole thing has changed and I’m experiencing it as new each and every time. This can be prompt huge anxiety and even a refusal to go (59).

Working on developing connection as monotropic people takes time and energy. It’s so much easier if we are understood and can understand and accept ourselves. Self-compassion is part of this journey. Technology can be helpful if we are people who are happy to work with smart phones, computers and tablets. These allow for reminders to be set, ways to respond that are not in person and so on.

## Conclusion

None of the above is easy or instant. It takes time to understand our autism (different for everyone) and it takes time to practice looking after our monotropic selves. Using strategies and accommodations we need to connect us to our interoceptive selves and to understand our OP challenges, will enable us to live the life we want. We do not live in a vacuum, and we do need the love and support from others. Self compassion though, is where we need to start. By accepting ‘the self’ we are born with and by loving and welcoming who we are we give ourselves permission to thrive. By actively incorporating into our daily lives those people, places, things we need and so on, to stay safe, connected and with autonomy, we will have all we need to live our best Autistic and authentic lives.

## Data Availability

The original contributions presented in the study are included in the article/supplementary material. Further inquiries can be directed to the corresponding author.
